# Altered Cell Wall Plasticity Can Restrict Plant Growth under Ammonium Nutrition

**DOI:** 10.3389/fpls.2017.01344

**Published:** 2017-08-10

**Authors:** Anna Podgórska, Maria Burian, Katarzyna Gieczewska, Monika Ostaszewska-Bugajska, Jacek Zebrowski, Danuta Solecka, Bożena Szal

**Affiliations:** ^1^Institute of Experimental Plant Biology and Biotechnology, Faculty of Biology, University of Warsaw Warsaw, Poland; ^2^Department of Plant Physiology, Institute of Biotechnology and Basic Science, University of Rzeszów Kolbuszowa, Poland

**Keywords:** ammonium toxicity syndrome, cell wall polysaccharides cross-linking, cell wall modifying enzymes, cell wall rigidity, growth inhibition, tensile stiffness

## Abstract

Plants mainly utilize inorganic forms of nitrogen (N), such as nitrate (NO_3_^–^) and ammonium (NH_4_^+^). However, the composition of the N source is important, because excess of NH_4_^+^ promotes morphological disorders. Plants cultured on NH_4_^+^ as the sole N source exhibit serious growth inhibition, commonly referred to as “ammonium toxicity syndrome.” NH_4_^+^-mediated suppression of growth may be attributable to both repression of cell elongation and reduction of cell division. The precondition for cell enlargement is the expansion of the cell wall, which requires the loosening of the cell wall polymers. Therefore, to understand how NH_4_^+^ nutrition may trigger growth retardation in plants, properties of their cell walls were analyzed. We found that *Arabidopsis thaliana* using NH_4_^+^ as the sole N source has smaller cells with relatively thicker cell walls. Moreover, cellulose, which is the main load-bearing polysaccharide revealed a denser assembly of microfibrils. Consequently, the leaf blade tissue showed elevated tensile strength and indicated higher cell wall stiffness. These changes might be related to changes in polysaccharide and ion content of cell walls. Further, NH_4_^+^ toxicity was associated with altered activities of cell wall modifying proteins. The lower activity and/or expression of pectin hydrolyzing enzymes and expansins might limit cell wall expansion. Additionally, the higher activity of cell wall peroxidases can lead to higher cross-linking of cell wall polymers. Overall, the NH_4_^+^-mediated inhibition of growth is related to a more rigid cell wall structure, which limits expansion of cells. The changes in cell wall composition were also indicated by decreased expression of *Feronia*, a receptor-like kinase involved in the control of cell wall extension.

## Introduction

The “ammonium syndrome” [symptoms of ammonium (NH_4_^+^) toxicity in plants, observed when using NH_4_^+^ as the sole nitrogen (N) source] is a global issue concerning most crop plants, with implications for agriculture, food industry, and environmental protection. Soil properties may determine the spatial distribution of plants and control ecosystem dynamics. Physiological and morphological disorders of plants, including leaf chlorosis, lower content of cations, lower root/shoot ratio, changes in amino acid or organic acid levels, and growth retardation (Gerendás et al., 1997; Walch-Liu et al., 2000; Britto and Kronzucker, 2002; Li et al., 2014) may be some of the reasons of agronomic problems, including lower yield or poor quality of food. The necessity of finding a method of alleviating this syndrome is one of the most important issues, particularly owing to anthropogenic effects on the global N cycle. Nitrogen is an essential macronutrient that is crucial for plant growth (Miller and Cramer, 2005), and N-use efficiency is a major factor determining plant biomass production (Kant et al., 2011; Xu et al., 2012). Nitrogen is the building brick for most organic molecules like amino acids, nucleic acids, or chlorophyll, and it is a constituent of the major electron transport nucleotides NADH and ATP. The utilization of NH_4_^+^ is bioenergetically more efficient than the other N sources. The assimilation of NH_4_^+^ to glutamine requires two electrons, whereas the reduction of NO_3_^–^ to NH_4_^+^ requires 10 electrons (Noctor and Foyer, 1998). Therefore, the sensitivity of most plants to NH_4_^+^ application as the sole N source, including effects such as severe growth suppression, is surprising.

Despite the numerous studies addressing the reasons for the symptoms of NH_4_^+^ toxicity in plants, the cause for this phenomenon has remained unclear. Most of these studies focused on intracellular metabolism. Ammonium is a photophosphorylation uncoupler, and NH_4_^+^ toxicity was attributable to the impairment of the photosynthetic processes (Raab and Terry, 1994; Gerendás et al., 1997; Bendixen et al., 2001). The strong demand of carbon skeletons in plant roots to participate in NH_4_^+^ assimilation was expected to deplete the carbohydrate supply to shoots (Schortemeyer et al., 1997; Walch-Liu et al., 2005, 2006). Direct competition between cation uptake and NH_4_^+^ influx, which employ common transport mechanisms, could induce deficiencies of essential cations, such as K^+^, Mg^2+^, and Ca^2+^ in plant tissues (Szczerba et al., 2008). The energetic cost of futile NH_4_^+^ cycling through the plasma membrane (i.e., NH_4_^+^ influx and removal in roots) is very high and may represent an energy burden for the whole plant (Britto et al., 2001; Kronzucker et al., 2001; Coskun et al., 2013). Moreover, NH_4_^+^ influx is accompanied by proton export, which may cause a transient alkalization of the cytosol (Britto and Kronzucker, 2005) and hyperacidification of the apoplast (Husted and Schjoerring, 1995). A potential disruption of the hormonal balance in plants growing on NH_4_^+^ might occur owing to the close linkage of phytohormones, including abscisic acid, auxin, and cytokinins, with NO_3_^–^ signaling (Cao et al., 1993; Walch-Liu et al., 2000). The disruption of redox homeostasis due to lower energy demand for NH_4_^+^ assimilation may cause oxidative stress (Podgórska et al., 2013). To date, the mechanism for the NH_4_^+^ toxicity syndrome is unknown, and therefore, it is not possible to alleviate NH_4_^+^-based growth inhibition.

It seems that in all present studies concerning ammonium toxicity one reason of growth retardation was omitted. Plant growth rate depends on cell division and/or cell elongation. One of the factors controlling these processes at the individual plant cell level is the presence of the cell wall. Plant cells are surrounded by the primary cell wall, a rigid structure that needs to be modified for allowing cell growth (Cosgrove, 2005). Primary cell walls of growing plants are mainly composed of three types of polysaccharides (cellulose, hemicellulose, and pectin), covalently bound ions (calcium and boron), and various proteins (e.g., structural glycoproteins, enzymes, and expansins). The major load-bearing cell wall polymer is the crystalline polysaccharide cellulose, made up of (1,4)-linked β-D-glucan residues. Cellulose forms microfibrils, which construct the skeleton of cell walls and provide mechanical strength. Hemicellulose forms branched chains containing mainly xyloglucan; these polymers bind to the surface of the cellulose microfibrils and hold them together. Pectins are a heterogeneous group of polysaccharides that contain galacturonic acid. These gel-forming polymers have the ability to form cross-links with the hemicellulose molecules of adjacent microfibrils. The emerging hemicellulose-pectin matrix supports the cellulose skeletal net; therefore, the cross-links between cellulose and non-cellulosic polysaccharides determine the rigidity of the cell wall (Park and Cosgrove, 2015). Some plant cells produce a secondary cell wall, which is stiffer owing to the cross-linking of hemicelluloses with lignins. Lignins are cell wall macromolecules that are made of highly cross-linked phenolic compounds.

All the constituents needed for cell wall assembly (except cellulose) are synthesized inside the cell in the Golgi apparatus and transported to the extracellular space, where they are incorporated in the cell wall network. However, cellulose is directly synthesized *in muro* by the cellulose synthase complexes, which are embedded in the plasma membrane (Somerville, 2006). The reorientation of cellulose synthase complexes guides cellulose microfibril deposition and facilitates cell expansion and growth. If the microfibrils are aligned in order, anisotropic growth is likely, and if the microfibrils are arranged randomly isotropic growth is possible. The architecture of the cell wall is essential for providing strength and structural support to the plant, and resisting turgor pressure. In addition, it has to be extensible for allowing cell growth. Therefore, the precondition for cell enlargement is cell wall loosening, which enables turgor-driven expansion of the cell wall. The principal enzymes and other proteins acting on cell wall assembly are: (A) xyloglucan endotransglucosylase/hydrolases (XTH/XETs), which integrate newly synthesized xyloglucan into the cell wall to strengthen the structure, (B) pectin hydrolyzing enzymes, e.g., endopolygalacturonases (PG), which can hydrolyze the pectin network, thereby leading to cell wall loosening; their action depends on de-esterification of pectins performed by pectin methylesterases (PME), (C) cell wall peroxidases (POX), which can catalyze the cross-linking of phenolics with hemicellulose and create the stiff secondary cell wall, and (D) expansins (EXP), which perform acid-induced wall stress relaxation due to non-enzymatic loosening of non-covalent adhesion between cellulose and hemicelluloses. However, which of these proteins are of greater importance for controlling the loosening of wall polymers during cell expansion in leaves is still debated. It is not plausible that only a single enzyme can be responsible for the changes in the cell wall mentioned above, because this process is stringently controlled and synchronized.

Remodeling of the cell wall is a fast mechanism that can regulate plant growth. Therefore, in recent years, the perception of the cell wall has changed to not being considered as dead matter, but an organelle actively reacting to diverse environmental stimuli, including mineral nutrition (Tenhaken, 2014; Le Gall et al., 2015). It was shown that NH_4_^+^-mediated suppression of growth might be attributable to both repression of cell elongation and reduction of cell division. Decreased leaf growth, indicated by smaller cell size of NH_4_^+^-treated tobacco, was related with decreased cell expansion and reduced cell number (Walch-Liu et al., 2000). Moreover, NH_4_^+^ triggered inhibition of primary root growth of *Arabidopsis thaliana* occurred by reducing the length of the meristem and elongation zone and decreasing the expansion rate of roots (Liu et al., 2013). Further, Li et al. (2010) claimed that reduced cell elongation and not cell division is the principal implication of NH_4_^+^-mediated inhibition of primary root growth of *Arabidopsis* seedlings. Because cell elongation depends on cell wall extensibility, and in turn on cell wall composition and spatial organization, the purpose of the present study was to determine the role of the cell wall structure on NH_4_^+^-triggered growth suppression.

## Materials and Methods

### Plant Material and Growth Conditions

*Arabidopsis thaliana* ecotype Columbia-0 were grown hydroponically using an Araponics growth system (Araponics, Liège, Belgium), as previously described in Podgórska et al. (2013). The plants were grown for 7 weeks continuously on nutrient medium containing Ca(NO_3_)_2_ (2.5 mM) or (NH_4_)_2_SO_4_ (2.5 mM) as the sole nitrogen source. NO_3_^–^-treated plants were considered as controls. Experiments were carried out on plant leaves.

### Leaf Blade Characterization

The growth parameters of leaves were monitored by measuring the area and the thickness of the ninth fully developed leaf. The area of the leaf blade was calculated using WinFOLIA leaf analysis software (Image Analysis for Plant Science, Regent Instruments Inc.). The thickness of the leaf blade samples (the mean for three locations) was determined using Nikon SMZ1000 (Japan) stereo microscope. The measurements were made using ImageJ (v.1.51f) software.

To gain insights into the mechanical properties of growing leaves we performed tensile tests on the leaf lamina in full turgor and rehydrated frozen-thawed samples. Leaf sections were cut (5 mm × 2 mm) from the front part of the leaves next to the central nerve, with a pair of razor blades mounted between a plastic separator. The leaf specimens were glued at the ends to plastic sticks with small amount cyanoacrylate adhesive. A half of the samples were examined for mechanical properties at full turgor and the other half was tested after freezing at -20°C for 1 h to destroy cell membrane. This procedure led to a complete reduction in turgor pressure of tissues so that the results depended only on the cell wall mechanical properties. Unidirectional tensile tests were performed on the computer-driven Instron universal machine (model 5542, High Wycombe, United Kingdom) at a deformation rate of 1 mm/min. The plastic sticks were firmly connected by means of grips to the load cell (10 N capacity) and to the immovable part of the instrument. Sample strains were measured locally at high accuracy (1/100 pixel) from video recordings analyzed using Video gauge system (Imetrum, United Kingdom). The displacement of selected points on the sample surface could be tracked at an accuracy level of approximately 10 μm for a gauge length of approximately 0.5 mm. The use of this non-contact video extensometer allowed us to avoid the interfering effects due to the sample slippage within clamps, and non-linearity in strain distribution close to the grids. The stress-strain curve was the basis for the calculation of mechanical parameters.

The relative strain (%) was calculated directly from the displacement of tracked points during tensile sample deformation. The strain at break was defined as (*x*_max_ – *x*_0_) ⋅ 100%/ x_0_ where *x*_0_ was the distance between the selected points before loading (the gauge length) and *x*_max_ was the distance between the same points at the break. The tensile stiffness EA of leaf structure was determined as the ratio of the load increment to the corresponding relative strain at the linear phase of sample deformation. The Young’s modulus (E) of the leaf blade was calculated as follows: *E = EA/A*, where EA is the tensile stiffness and A is the cross-sectional area of the sample. The tensile strength was defined as the ratio of the load at break to the value of A.

### Cell Wall Preparation and Protein Extraction

Cell walls were prepared according to the method described by Solecka et al. (2008). Isolated cell walls were utilized for total polysaccharide determination, cell wall fractionation and protein extraction for enzymatic measurements. Cell walls were extracted from around 5 g of frozen leaf tissue in 0.05 M HEPES-KOH, pH 6.5 and centrifuged at 5,000 × *g* for 10 min. The pellet was washed three times with cold water. Proteins from the crude cell wall preparations were extracted using 1 mL 0.05 M HEPES, 1 M NaCl, and 1 dose of protease inhibitor cocktail (Complete Ultra Mini EDTA-free Easy pack, Roche, Penzberg, Germany) under continuous agitation at 4°C for 12 h. The homogenates were centrifuged at 13,000 × *g* for 15 min and the resulting supernatant containing cell wall proteins was recovered, whereas the pellet collected was comprised of cell walls. The cell wall residue was washed three times with water, twice with 70% (v/v) ethanol at 70°C, and 100% acetone. The resulting precipitate containing the cell wall was air dried for 24 h and used for cellulose, pectin and lignin determination. The supernatant was desalted and concentrated using centrifugal filter devices (10K cut-off), according to the manufacturer’s protocol (Microcon; Millipore, Billerica, MA, United States). The recovered supernatant that had not passed through the filter contained the extracted cell wall proteins. Soluble protein content in cell wall extracts was estimated using the colorimetric method of Bradford (1976).

The cell wall residue was further fractionated into the pectin-enriched fraction and a hemicellulose-enriched fraction. A washing step with 90% (v/v) dimethyl sulfoxide (DMSO) for 24 h at RT was applied to eliminate starch contamination in cell walls (Kubacka-Zęebalska and Kacperska, 1999). The resulting pellet was washed free of residual DMSO by several washings with 96% (v/v) ethanol and evaporated to dryness. The cell wall preparations were extracted with 10 mL of 0.5% (v/v) ammonium oxalate at 100°C for 1 h. The ammonium oxalate soluble fraction was dialysed for 50 h against deionised water, evaporated to dryness under reduced pressure and weighed. This dry mass represented the pectin-enriched fraction. The ammonium oxalate insoluble pellet was dissolved in 10 mL 4 M KOH supplemented with 20 mM NaBH_4_ for 12 h at RT. The alkaline-soluble fraction was acidified to pH 5.5 with acetic acid, dialysed for 50 h against deionised water, evaporated to dryness under reduced pressure and weighed. This dry matter represented the hemicellulose-enriched fraction. The obtained fractions were utilized for uronic acids and neutral sugar determination and FTIR analysis.

### Polysaccharide Quantification in Cell Walls

Total cellulose content of the extracted cell walls was measured using the colorimetric Anthrone assay, according to the method of Updegraff (1969). All the polysaccharides, except crystalline cellulose, were released from the cell walls (10 mg) by incubating dry material in a 1 mL mixture of HNO_3_ and CH_3_COOH (1:8) for 1 h at 100°C. The homogenate was centrifuged at 12,500 × *g* for 10 min and the pellet was washed twice with water. The resulting pellet containing cellulose was dissolved in 1 mL 67% H_2_SO_4_ (v/v). The samples were transferred to 0.8 mL 0.2% cold Anthrone reagent in concentrated H_2_SO_4_. After boiling for 5 min, blue color development was observed. Cellulose content was measured spectrophotometrically at 620 nm against cold Anthrone reagent. The results were presented as equivalents of glucose used in the standard curve.

Pectins were estimated as uronic acid content in cell walls and in the pectin-enriched fraction, according to the colorimetric method established by Blumenkrantz and Asboe-Hansen (1973). The samples were dissolved in water and digested with 1.2 mL 0.0125 M Na_2_B_4_O_7_ in concentrated H_2_SO_4_ at 100°C for 5 min. After cooling the samples, 20 μL 0.15% meta-hydroxybiphenyl in 0.5% NaOH was added for pink color development. Pectin content was estimated spectrophotometrically at 520 nm against blank samples. The results were presented as equivalents of galacturonic acid used as the standard.

Neutral sugar content was measured in the pectin-enriched fraction, according to the phenol sulfuric acid method of Dubois et al. (1956). The samples (2 mg) were diluted in water and hydrolyzed in 300 μL concentrated H_2_SO_4_, followed by addition of 50 μL 5% phenol. After 30 min incubation at room-temperature (RT), yellow color development was observed and absorbance was measured at 490 nm against blank sample. The neutral sugar content was presented as equivalents of glucose used as the standard.

Total lignin content in cell walls was determined by the acetyl-bromide method (Johnson et al., 1961) as described in Hatfield et al. (1999). Dry cell wall material (approximately 20 mg) was dissolved in 1 mL 25% acetyl-bromide in acetic acid for 2 h at 50°C. The samples were further dissolved in 5 mL 2 M NaOH, 0.75 M hydroxylamine-HCl, diluted with acetic acid and incubated overnight at RT. Lignin content was measured spectrophotometrically at 280 nm; the results were presented as equivalents of coniferyl alcohol.

### Ion Content Determination in Plant Tissues

Boron and Ca^2+^ concentrations in absolute dry mass samples from leaves were determined by Inductively Coupled Plasma Optical Emission Spectrometry (ICP-OES). The analysis was performed in an authorized Laboratory of Quality Investigation of Horticultural Products at the Horticultural Research Institute (Skierniewice, Poland).

### Phenol Estimation

The amount of phenolics bound to cell walls was measured using the Folin method (Forrest and Bendall, 1969) as described earlier in Solecka et al. (1999). Phenolics were released from the cell wall preparations by alkaline hydrolysis. Extractions were carried out by vortexing samples in 0.4 mL 0.5 M NaOH and incubating for 24 h at RT. After neutralization with HCl, the homogenates were centrifuged at 13,000 × *g* for 20 min. The supernatants containing phenols were added to 0.2 N Folin reagents (Sigma). Thereafter, 0.6 mL 7.5% Na_2_CO_3_ was added and samples were incubated for 30 min at RT in darkness. The phenolic content was determined spectrophotometrically at 750 nm against a blank sample; ferulic acid was used as a standard.

### Enzyme Activity Measurement

Enzymatic activity of pectinases was determined in cell wall protein extracts. The activity of PME was estimated following the colorimetric method described by Solecka et al. (2008). The reaction mixture was composed of 0.2 M NaCl, 0.015% (w/v) Methyl Red as a pH indicator, and contained 0.5% citrus pectins as the substrate for PME with different grades of methylesterification (8.9, 30, and 90%, respectively). The pH of the reaction mixture was titrated to three different pH values: 5.0, 6.8, and 8.5. The reaction performed by PME was traced after addition of 50 μL of the cell wall protein extracts, and the changes in color from yellow to red were detected using a spectrophotometer at 525 nm. A calibration curve was obtained by adding 1–200 nEq H^+^ to the reaction mixture.

The activity of PG was determined using the spectrophotometric method of Gross (1982) with minor modifications. The reaction was induced by adding 0.5% polygalacturonic acid in 50 mM sodium acetate buffer (pH 5.2) to cell wall protein extracts and incubated at 37°C for 2 h. The reaction mixture was diluted with 50 mM sodium acetate buffer (pH 5.2), and 30 μl cyanoacetamide was added to label the released reducing end-groups. After inactivation of the enzymes by boiling for 10 min, the absorbance was recorded at 320 nm. The activity was calculated based on galacturonic acid released by PG.

The activity of enzymes engaged in cell wall lignification was determined in cell wall protein extracts. POX activity was monitored spectrophotometrically by using guaiacol as the substrate (Veljovic-Jovanovic et al., 2001).

The activity of phenylalanine ammonia-lyase (PAL) was measured using the spectrophotometric method by Havir and Hanson (1968), as described by Solecka and Kacperska (1995). The reaction mixture was composed of 0.016 mM L-phenylalanine, 0.15 mM borate buffer (pH 8.8), 3.6 mM NaCl, and 50 μL of extracted proteins. Production of cinnamic acid was monitored after incubation of the samples at 37°C for 3 h. The enzymatic reaction was inhibited by adding 500 μL 6 M HCl and the increase in absorbance was measured at 290 nm against a blank sample. To eliminate non-specific reactions during the incubation time, additional tests were performed using reactions without the addition of L-phenylalanine as the substrate, or with extracts deactivated by boiling.

### Immuno-Blotting Analysis

To determine XTH/XET protein levels, 20 μg of cell wall proteins were separated in 12% sodium dodecyl sulphate (SDS)–polyacrylamide gel electrophoresis. Anti-XTH/XET (Agrisera, Vännäs, Sweden) were used as primary antibodies (diluted 1/500) and anti-mouse antibodies (Bio-Rad, Hercules, CA, United States) were used as the secondary antibodies. Immuno-blotting was performed according to the standard protocol. Bands for XTH/XET located at 33 kDa were determined on the basis of a pre-stained protein marker (Bio-Rad) as the reference. The relative protein levels were quantified by densitometry analysis using Image-Lab 5.2. software (Bio-Rad).

### Infrared Spectroscopy

Mid-infrared spectroscopy measurements were performed on cell wall material ground into a fine powder with an agate mortar and pestle and washed successively with 70% ethanol, 1:1 (v/v) mixture of chloroform and methanol, and acetone. Spectral data were collected using Fourier Transform Infrared (FTIR) spectrometer (Nicolet iZ 10 module, Thermo Fisher Scientific Inc., United States) equipped with a deuterated triglycine sulfate (DTGS) detector, KBr beam splitter, and a diamond single bounce Attenuated total reflectance (ATR) accessory. Two-hundred and fifty-six interferograms were recorded and added together between 4,000 and 400 cm^-1^ at 2 cm^-1^ resolution. The spectra were baseline corrected, normalized to a constant area between 1,760 and 880 cm^-1^, and smoothed at a final resolution of 4 cm^-1^ by apodization using the Blackman–Harris 3-term function and a zero-filling factor of 2. Collection, pre-processing, and analysis of spectral data were performed by means of OMNIC software (v. 9.0, Thermo Fisher Scientific Inc.). The spectra were subjected to multiple univariate and multivariate analysis. The latter was performed by unsupervised principal component analysis (PCA) (Unscrambler X, v.10.1, CAMO, Norway).

### Atomic-Force Microscopy of Cell Walls

The cell walls were isolated and purified to reveal cellulose microfibrils according to the method of Davies and Harris (2003), with minor modifications. The leaf tissues were homogenized in liquid nitrogen, and aliquots of the resulting powder were homogenized in 1% SDS, three times. The resulting residue was incubated successively with 50 mM Trans-1,2-diaminocyclohexane N,N,N′,N′-tetraacetic acid (CDTA, adjusted to pH 7.0 with KOH) at 70°C for 30 min (three times) and 50 mM Na_2_CO_3_ overnight at 4°C to wash out pectic polysaccharides. Finally, the cell wall deposit was washed three times with warm water. After each incubation, the suspension was centrifuged (5,000 × *g* for 10 min) and the supernatant was discarded. The final pellet containing cell wall probes was resuspended in water and used for Atomic Force Microscopy analysis (AFM).

AFM measurements were done with an Agilent 5500 atomic force microscope (Agilent, United States) in contact mode. The 205 nm long triangular cantilever B of SNL-10 Bruker AFM probe (Bruker Probes, United States), with 2 nm tip radius was used with force const. of 0.12 N/m. Aqueous dispersions of the cell wall were layered on a freshly cleaved mica (SPI Supplies, United States) and then allowed to dry in air. Imaging was carried out in the air whilst the samples remained visibly moist. If possible images were recorded at a resolution of 256 × 256 or 512 × 512 and 1 ln/s scanning speed. Topography, phase, and amplitude images were recorded with PicoView software (Agilent, United States) and analyzed with Gwyddion software (Nečas and Klapetek, 2012). The images were flattened to remove tilt or bow in each scan lie and exported to TIFF format.

### Determination of Cell Wall Ultrastructure

Ultrastructural imaging of mesophyll leaf cells were performed as described in Podgórska et al. (2013), and the thickness of the cell walls was measured in micrographs obtained by transmission electron microscopy (TEM). Briefly, small sections of plant leaves were fixed in 2.5% (v/v) glutaraldehyde in 50 mM sodium cacodylate buffer (pH 7.3) for 4 h at RT. The tissues were post-fixed in 2% (v/v) OsO_4_ solution in 50 mM sodium cacodylate buffer (pH 7.3) overnight at 4°C. Thereafter, the samples were stained in 1% (w/v) uranyl acetate in 70% (v/v) ethanol for 1 h. This was followed by further dehydration in a graded ethanol series (70–100%) and acetone series up to 100% acetone. The plant material was progressively fixed in Epoxy embedding medium. The tissues were polymerized in 100% EPON at 60°C for 72 h. The blocks containing the embedded sample were sectioned (90 nm) on a RMC PowerTome microtome (Boeckeler Instruments, Tucson, AZ, United States) and mounted on uncoated copper grids (300 meshes). The sections were examined using a JEM 1400 transmission electron microscope (JEOL Co., Japan) and a high-resolution digital camera (CCD MORADA, SiS-Olympus, Germany) at the Laboratory of Electron Microscopy, Nencki Institute of Experimental Biology, Warsaw, Poland. The thickness of a double layer of cell walls was determined in micrographs using Image Processing and Analysis in Java software (ImageJ). The measured zone included the primary cell wall and the middle lamella of two adjacent cells.

### Polysaccharides Localization by Confocal Microscopy

For determining cellulose localization, the upper epidermis was removed from fresh plant leaves and the tissues were cut into small sections (approximately 5 mm). The samples were stained with 0.5 mg/mL Calcofluor White (Sigma) for 5 min at RT. The leaf pieces were washed in water three times and placed on a glass slide. A NIKON A1R MP confocal laser scanning system (Zeiss LSM-510) was used to detect fluorescence with a 60× (numerical aperture 1.2) water immersion objective. Calcofluor White fluorescence was induced with the 404 nm helium-neon laser at an emission of 490 nm. In these micrographs, the cell diameters were determined, measuring the length and width of each cell. The resulting cell area was calculated assuming an ellipsoid cell shape, using the following formula: *A =* π*ab*, where *a* is the leaf length and *b* the leaf width, and A is the cell area.

The localization pattern of pectins and xyloglucan in leaf tissues was studied using monoclonal antibodies, which bind to specific cell wall epitopes. Hand-cut sections of the leaf stalk and the leaf blade were fixed in 0.5% glutaraldehyde with 4% paraformaldehyde in sodium phosphate buffer (pH 7.4) for 2 h. Thereafter, the tissues were washed three times in sodium phosphate buffer for 10 min and dehydrated in a graded ethanol series of 30–100% (v/v), for 10 min in each grade. Further, the specimens were progressively fixed in Steedman’s wax dissolved in ethanol at 40°C. The plant material was polymerized in 100% Steedman’s wax at RT. The wax blocks containing the embedded samples were sectioned (20 μm) on a RMC PowerTome microtome and mounted on Poly-L-Lysine coated glass slides. After rehydratation of the samples in decreasing ethanol concentrations of 96–30% (v/v), slides were used for immunolocalization. Also fresh leaf blades were embedded in 4% agar in 0.9% NaCl and sectioned (150 μm) using a vibratome. The fixed specimens and fresh leaf sections were washed with tris-buffered saline (TTBS) buffer and incubated with a blocking buffer containing: 5% bovine serum albumin (BSA) in TTBS and 0.1% Tween20 for 60 min at RT. The sections were immunolabelled with the primary-antibodies LM19, LM20 or LM25 (1:50, Plant Probes, Inc., United Kingdom) in 0.5% BSA in TTBS overnight at 4°C with gentle shaking. After six washes with TTBS buffer, a secondary antibody (1:100, Anti-rat-Alexa IgM, Fluor 488, Molecular Probes, Leeds, United Kingdom) was applied for 1 h at RT. Finally, the sections were rinsed with TTBS buffer and mounted with Vectashield (Vector, United States) anti-fading medium. The fluorescence was observed with NIKON A1R MP confocal laser scanning system (Zeiss LSM-510) at 10× and 60× magnifications and photographed. The fluorescence was measured using the 488 nm line of an argon-krypton laser and the emission was recorded for 500–545 nm. Negative controls were performed by omitting the primary antibody step, obtaining no fluorescence signal in each control frame for all slides stained.

### Quantitative Real-Time Polymerase Chain Reaction

The mRNA level of cell wall enzymes was quantified by real-time polymerase chain reaction (qRT-PCR) analysis. Total RNA was extracted using the RNeasy Plant Mini kit (Qiagen, Hilden, Germany) according to the manufacturer’s protocol. First strand cDNA synthesis was performed using 1 μg total RNA as a template with reverse transcriptase following the RevertAid H minus first strand cDNA synthesis kit (Fermentas, Thermo Fisher Scientific, Inc., Waltham, MA, United States), according to the manufacturer’s protocol. Thereafter RNA digestion was performed as described by Escobar et al. (2004). For all qRT-PCRs, the iTaq SYBR Green supermix was used (Bio-Rad) at 2× diluted reactions. New primer pairs were designed, and the sequence of at least one of the primer pairs covered an exon-exon border. The primer sequences were as follows: *FER* (At3g51550): 5′-TGCCGTCACTTCTCGTTTGC-3′ and 5′-CTTGCTCGGACATTGGGTTG-3′; *THE1* (At5g54380): 5′-TCAAGAAGGCGGTAATGGACA-3′ and 5′-CAACCCCAAGCAACGAACTC-3′; *EXP1* (At1g69530): 5′-GAAGAGTGCCGTGCGTGAG-3′ and 5′-TAGGTTGAAGTAAGAGTGTCCGTTT-3′; *EXP17* (At4g01630): 5′-GCCTCTGGTACAATGGGTGG-3′ and 5′-TGAGCAAAGTTCGGTGGACA-3′; *PG1* (At3g26610): 5′-CCAATGAAACCAACGGCACT-3′ and 5′-TGACTTTGACTCCATCCGAATC-3′; *Putative-PG* (At2g43890): 5′-ACAGGGTTCTGGAGTGAAGATTAGTC-3′ and 5′-TCGCTTGGCACGGATTACT-3′.

The qRT-PCRs were performed using a thermo cycler (IQ5 RT-PCR detection system, Bio-Rad), using 60°C as the annealing temperature. Transcript abundance was quantified by comparing the quantification cycle (*C*q) values of the target gene relative to those of the reference gene *PP2A* (At1g13320, Czechowski et al., 2005) according to the method described by Pfaffl (2001). The transcript levels of each gene were expressed in relation to those in control plants (set as 1).

### Statistical Analysis

The results were expressed as the mean values ± standard deviations from 3 to 20 measurements taken from 3 to 5 independent plant cultures. Experimental data from plants grown on NO_3_^–^ or NH_4_^+^ was statistically analyzed with Student’s *t*-test using Microsoft Excel (Microsoft). Results shown with an asterisk were significantly different at a *p*-value < 0.05.

## Results

### Ammonium-Based Plant Growth Characterization and Mechanical Properties of Tissues

**FIGURE 1:**
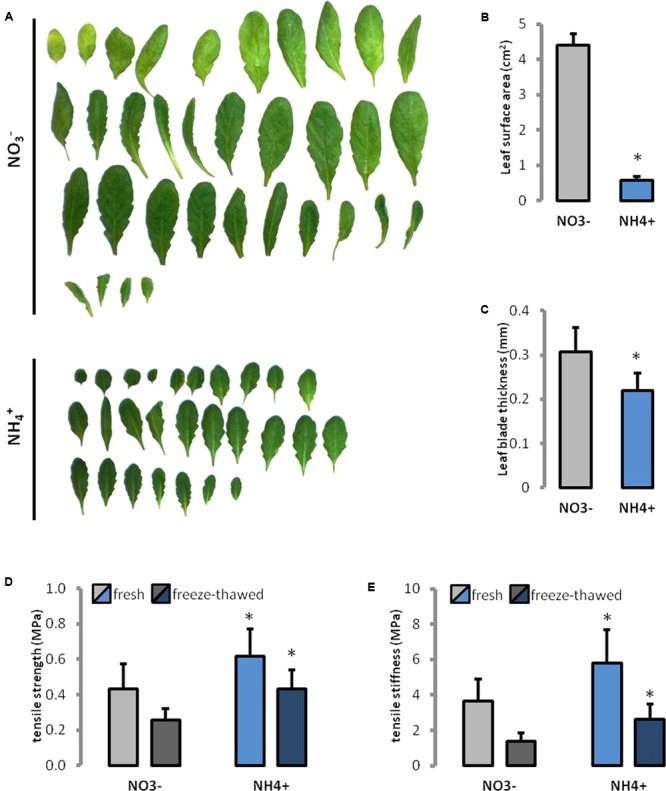
Leaf phenotype recorded in *Arabidopsis thaliana* growing on NH_4_^+^ and NO_3_^–^ (control) as the only N source. **(A)** Leaves were ordered from the oldest to the youngest. **(B)** Leaf area, and **(C)** thickness of the leaf lamina of the ninth fully expanded leaf. Mechanical properties of leaves, **(D)** the tensile strength, and **(E)** tensile stiffness were measured in fully hydrated fresh leaves and in rehydrated frozen-thawed (1 h at –20°C) tissues.

During NH_4_^+^ nutrition plants showed overall smaller leaves in their rosettes than NO_3_^–^ treated controls (**Figure 1A**). The leaf area was seven-times smaller during growth on NH_4_^+^ than on NO_3_^–^ (**Figure 1B**). Further, the thickness of the leaf lamella was 30% less in plants grown on NH_4_^+^ as the only N source (**Figure 1C**).

The maximum stress that could be borne by the tissues (tensile strength) increased by more than 40% in fresh leaves and 70% in rehydrated frozen-thawed leaves of plants grown on NH_4_^+^ than in the controls (**Figure 1D**). The increase in tensile stiffness (the effective Young’s modulus) of the leaf tissue was about 60 and 90% higher in fresh and rehydrated frozen-thawed leaves, respectively, than in the control (**Figure 1E**). Additionally, in plants grown on NH_4_^+^ a higher ratio of the stiffness to the strength of fresh leaves (9.4 vs. 8.5) and for cell-wall preparations obtained after freezing and thawing (6.07 vs. 5.45).

### Changes in Cell Wall Morphology

**FIGURE 2:**
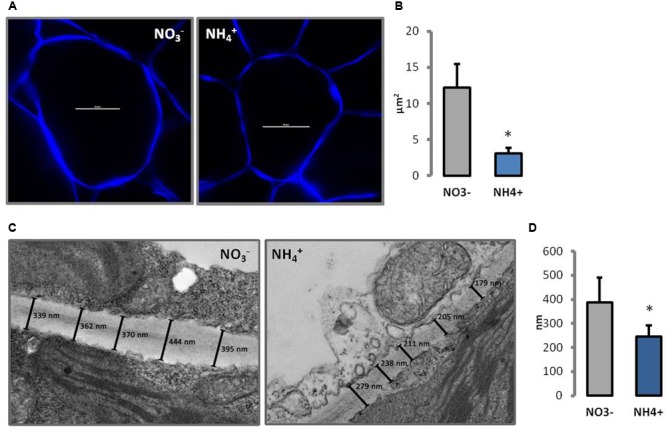
Cell wall morphology of leaf mesophyll cells of plants growing on NH_4_^+^ and NO_3_^–^ (control) as the only N source. **(A)** Cell wall visualization of individual palisade cells by Calcofluor White staining in CLSM (scale bar = 20 μm), and **(B)** cell size measurement from six independent biological replicates. **(C)** Ultrastructure of cell walls. The lines indicate the measured points in the cell wall. A representative of the 20 independent images that were used for measuring cell wall thickness **(D)**.

Changes in morphology of cell wall were observed in CLSM and TEM. In transverse sections through leaf cell walls, the middle lamella and primary cell wall were identified and the thickness of both layers of the cell wall was determined. The cell walls of NH_4_^+^ grown plants were around 40% thinner than in the controls (**Figure 2D**). There were no other changes in leaf cell wall ultrastructure seen in response to the N treatments (**Figure 2C**). In addition, the cell walls were traced *in vivo* using fluorescent dyes. Calcofluor White is a stain that reveals all-â-glucan-containing cell walls. Therefore, it is highly effective in visualizing cell wall disassembly. Transverse sections of palisade mesophyll leaf cells revealed slightly thinner cell wall of plants grown on NH_4_^+^ than in the controls (**Figure 2A**). Moreover, it was noticed that the cells of NH_4_^+^-treated plants were smaller. The size of individual cells in the leaf blade was up to five times lower in plants growing on NH_4_^+^ than in controls (**Figure 2B**).

**FIGURE 3:**
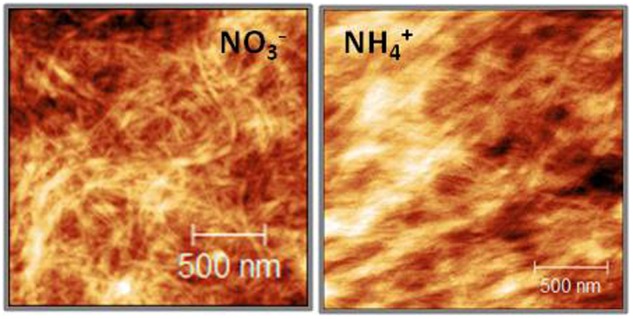
Atomic force micrograph (height mode image) of partially hydrated, isolated cell walls of *A. thaliana* leaves of plants growing on NH_4_^+^ and NO_3_^–^ (control) as the only N source. All images are 2 μm × 2 μm.

The AFM technique is widely used to study cell walls revealing layered fibrous structures displaying microfibril arrangement (Kirby et al., 1996, 2006; Kirby, 2011; Posé et al., 2012). The rigidity of the cellulose network allows imaging in direct contact between AFM probe and cell wall surface even in the air (Kirby et al., 1996). The bright and dark areas in the topography image correspond to peaks and troughs of the sample surface. A fibrous structure can be seen in both variants (**Figure 3**). The fibers in cell walls from NH_4_^+^ grown plants are more tightly arranged, they run in the same direction, whereas in the case of the NO_3_^–^ treatment the fibers are crooked and scattered.

### Changes in Cell Wall Composition

**FIGURE 4:**
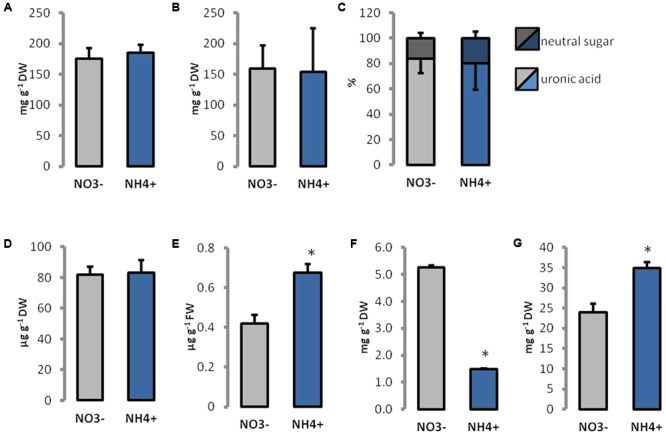
Content of major polysaccharides and other components in cell wall constituents isolated from leaves of plants growing on NH_4_^+^ and NO_3_^–^ (control) as the only N source. **(A)** Cellulose and **(B)** pectin levels were measured in dried cell walls. **(C)** The proportion of uronic acids and neutral sugars in the pectin-enriched fraction. The content of **(D)** lignin and **(E)** phenolics isolated from cell walls. **(F)** The content of calcium and **(G)** boron ions in leaf tissues.

Ammonium treatment did not affect the level of cellulose, pectins, and lignin in cell walls (**Figures 4A,B,D**). Further the content of homogalacturonan and neutral sugars in the pectin-enriched fraction showed no statistically relevant changes (**Figure 4C**), but a slightly higher percentage of neutral sugars in cell walls of NH_4_^+^ grown plants could be observed. The total content of phenolics in the cell wall was more than 60% higher in leaves of NH_4_^+^ treated plants than in the controls (**Figure 4E**). More than 70% lower content of Ca^2+^, but 45% higher content of boron was detected in tissues of NH_4_^+^-treated plants than in the controls (**Figures 4F,G**).

**FIGURE 5:**
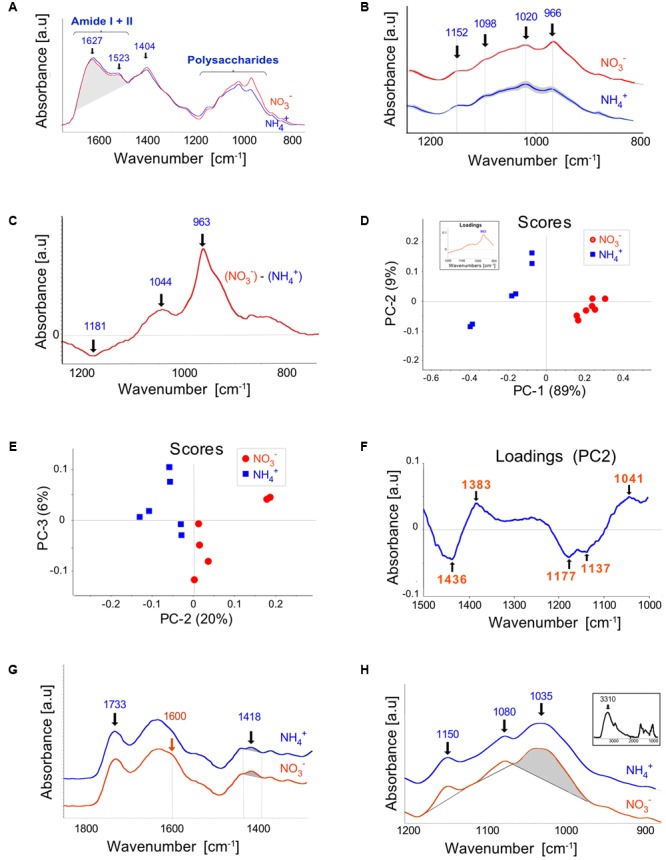
Fourier Transform Infrared (FTIR) spectroscopy of cell walls extracted from leaves of *Arabidopsis* plants cultured on NH_4_^+^ and NO_3_^–^ (control). **(A)** The spectra for the Amide I + II and the fingerprint regions normalized for an equal area between 1,760 and 880 cm^-1^. **(B)** The mean spectra with SD (gray ribbon) for the polysaccharide region. For better readability, they were offset along the absorbance axis. **(C)** Differential mean spectrum of the cell wall obtained by subtraction the spectrum for plants grown on NH_4_^+^ and NO_3_^–^. **(D,E)** PCA score plots discriminating plants growing on NH_4_^+^ and NO_3_^–^. Principal component 1 (PC1) explained 89% of the total variability within the spectral range between 1,200 and 900 cm^-1^ and PC2 explained 20% of the variability between 1500 and 1000 cm^-1^. Corresponding loadings are given as an inset in **(D)** and as the **(F)**. Infrared spectra of **(G)** pectin-enriched and **(H)** hemicellulose-enriched extracts, respectively. The inset in **(H)** shows the whole-range spectrum for hemicellulose-enriched extracts. The relevant wavenumbers are denoted in the figures or shaded. The means were calculated for three independent experiments and two technical replicates.

Infrared spectra (1,800–800 cm^-1^) obtained by FTIR analysis of cell walls isolated from leaves of plants grown on NO_3_^–^ and NH_4_^+^ nutrients revealed that the amide I + II region (1,700–1,480 cm^-1^) characterized by peaks at 1,627 and 1,523 cm^-1^ did not differ significantly in area as well as in the shape (**Figure 5A**), thereby indicating the lack of striking evidence for alteration in the structural proteins owing to the N source used. The band centered at 1,627 can be ascribed to C=O stretching in the COO^-^ group of non-methylesterified pectins (Jones et al., 2005). However, the presence of absorbance in the region near 1,523 cm^-1^, which is generally absent from the spectra of pectins, evidences the contribution of proteins in the Amide I band. The most prominent FTIR spectral dissimilarities between the plant nutrition variants were observed in the polysaccharide region between 1,200 and 900 cm^-1^ (**Figure 5B**). The major bands located at 1,152, 1,098, and 1,020 cm^-1^, can be assigned to C-O-C asymmetric stretching, and C-C/C-O stretching vibrations, respectively. The integrated peak area was lower for the bands at 1,098 and 1,020 cm^-1^ in NH_4_^+^- than in NO_3_^–^-treated plants. Differential (NO_3_^–^-NH_4_^+^) spectra (**Figure 5C**) provided additionally a positive peak at 1,044 cm^-1^ and a negative peak at 1,181 cm^-1^, assigned to C-O and C-C stretching in the ring of xyloglucan (Kačuráková et al., 2000) and to C-O stretching and/or guaiacyl ring breathing, respectively. Unsupervised multivariate analysis (PCA) confirmed that spectral features in the polysaccharide region distinctly separated the NH_4_^+^ and NO_3_^–^-treated plants in the clustering pattern (**Figure 5D**). Plants grown on NH_4_^+^ showed negative, whereas NO_3_^–^-treated plants positive scores for the principal component (PC) 1, explaining 89% of the total spectral variability. The PC1 loading factor plot (inset in **Figure 5D**) showed positive association with peak at 963 cm^-1^, additionally confirming its discriminating role between the NH_4_^+^ and NO_3_^–^-treated plants (**Figure 5C**). This band, however, could not be unequivocally assigned. This band was present both in the NH_4_^+^ and NO_3_^–^-treated plants and was markedly elevated in the latter.

Because the peak at 963 cm^-1^ dominated the spectral variability within the polysaccharide range and thus could mask the contribution of other compounds we performed also the PCA for the shifted range of spectra between 1500 and 1000 cm^-1^. This spectral region also discriminated the nutrient treatments as revealed by the score plot in **Figure 5E**. The corresponding loadings (**Figure 5F**) showed negative correlation for the bands between 1180 and 1130 cm^-1^, putatively assigned to C-O stretching of C-OH groups and to C-O-C stretching of glycosidic links and/or to ether-linked phenolic compound. Since the loading feature did not resemble that typical for polysaccharides this data may indicate increased amount of ether-linked phenolic compounds in NH_4_^+^ plants compared to the NO_3_^–^ control.

To estimate possible differences in the degree of esterification of pectins, FTIR spectra were performed on the pectin-enriched fraction (**Figure 5G**). The carbonyl C=O stretching vibration of the ester group at about 1735 cm^-1^ (McCann et al., 2001) was characterized by similar absorbance (normalized to the area between 1800 and 900 cm^-1^) for both nutrient variants. The NO_3_^–^ grown plants showed a slightly more visible shoulder about 1600 cm^-1^ and higher absorbance at 1418 cm^-1^, corresponding to vibrations of COO-group of polygalacturonic acids (Séné et al., 1994; Jones et al., 2005). However, the ratio of the absorbance at 1600 to 1730 cm^-1^, that is indicative of the degree of pectin esterification (Filippov and Kohn, 1975), did not differ significantly. The analysis of the hemicellulose-enriched fraction (**Figure 5H**) showed bands at 1150, 1080 and 1035 cm^-1^, the latter two are tentatively due to complex C-OH, C-O-C and C-C stretching vibrations, which may be attributed to xyloglucans (Kačuráková et al., 2000). The absorbance area of the band centered at 1035 cm^-1^, normalized to the height of the maximum peak for the whole spectrum at 3310 cm^-1^ (**Figure 5H**, inset) was higher for the NH_4_^+^ treated plants compared to NO_3_^–^ grown controls (0.0301 vs. 0.0285, respectively), the difference was statistically not significant though. Some interfering impact due to presence of arabinogalactans to the absorbances cannot be excluded.

### Immunolocalization of Cell Wall Polysaccharides in Leaves of NH_4_^+^-Treated Plants

**FIGURE 6:**
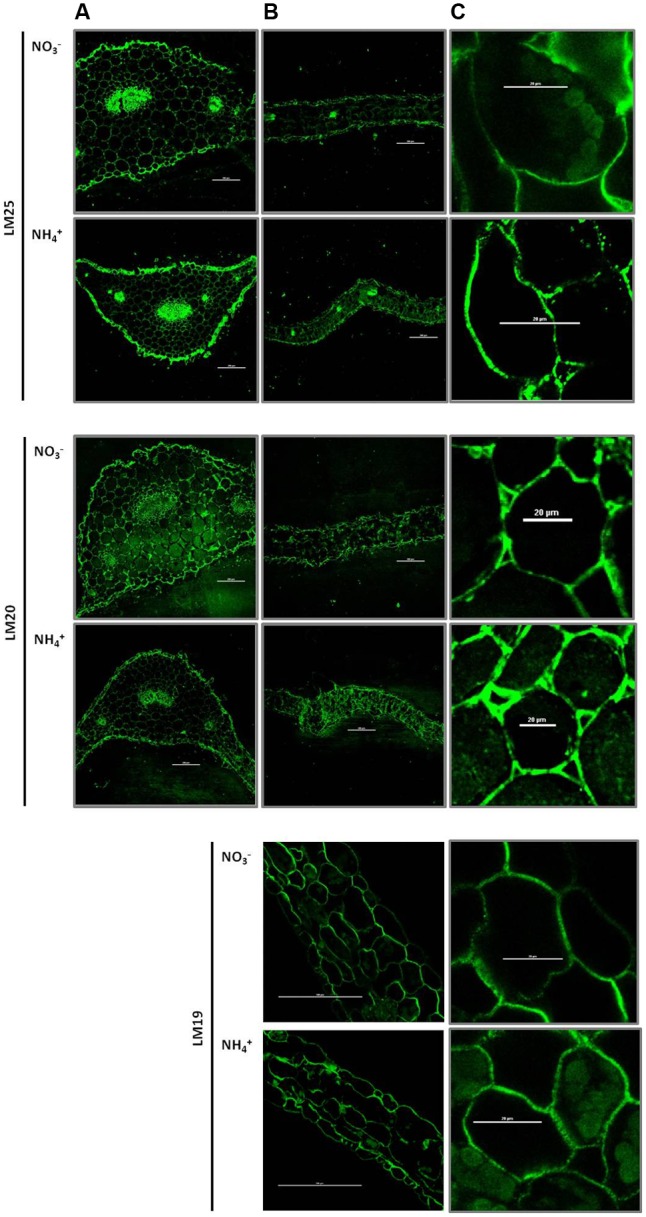
Immunolocalization of polysaccharide epitopes in plants growing on NH_4_^+^ and NO_3_^–^. Distribution of xyloglucan in cell walls, sections labeled with LM25 antibody. Distribution of highly methylesterified homogalacturonans in cell walls – labeled with the LM20 antibody and low-methylesterified homogalacturonans – labeled with the LM19 antibody. Immunocytochemistry of plant cell walls was performed in the **(A)** petiole (scale bar = 200 μm), **(B)** leaf blade (scale bar = 200 μm) and **(C)** a close up of a leaf cell (scale bar = 20 μm) was performed. Representative results from four independent biological replicates are shown.

The major cell wall hemicelluloses are xyloglucan. Immunofluorescence studies displayed the xyloglucan-specific motifs in cell walls recognized by the monoclonal antibody LM25. The level of xyloglucan, indicated by green fluorescence of the LM25-treated sections, was higher in mesophyll leaf cells from NH_4_^+^-treated plants than in the controls (**Figures 6B,C**). In addition, a higher fluorescence signal was observed preferentially in the epidermis of the petiole in NH_4_^+^ grown plants (**Figure 6A**). Regions of highly methylesterified pectins in cell walls were labeled by the LM20 antibody-dependent fluorescence. The LM20 dependent labeling was relatively high in mesophyll leaf tissues during NH_4_^+^ nutrition than in the control (**Figures 6B,C**). Fluorescence showed a similar pattern in the petiole between different N treatments (**Figure 6A**). Also epitopes of low-methylesterified pectins in cell walls were labeled by the LM19 antibody. The resulting fluorescence in leaf cells showed a similar intensity between different nitrogen growth regimes (**Figures 6B,C**). Negative controls (fixed micro-sliced sections of *Arabidopsis* leaves treated with secondary antibodies) showed only lignin autofluorescence.

### Changes in Cell Wall Modifying Proteins

**FIGURE 7:**
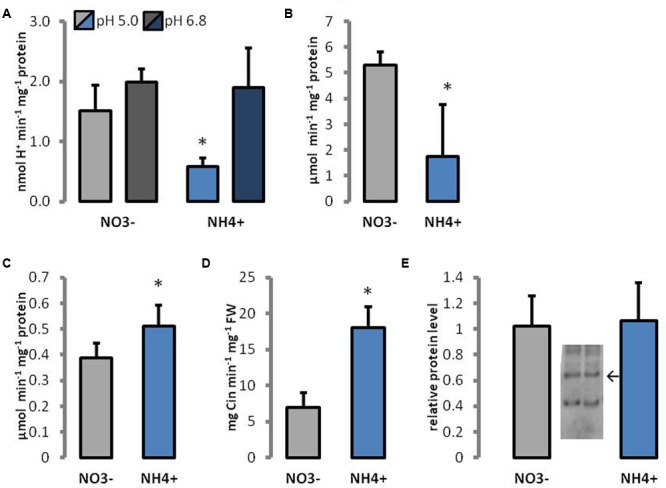
Enzymes engaged in cell wall metabolism of plants growing on NH_4_^+^ and NO_3_^–^ (control). The activity of **(A)** pectin methylesterases, **(B)** polygalacturonases, **(C)** cell wall peroxidases, and **(D)** phenylalanine ammonia lyases, in leaf tissues, and **(E)** the protein level of xyloglucan endotransglycosylases; arrow indicates the band representing 33 kDa specific for XTH/XET. Signal intensities of bands were estimated using Image-Lab 5.2 software. Representative results from four independent biological replicates are shown.

Activities of cell wall enzymes engaged in pectin hydrolysis were determined. PME is responsible for demethylesterification of pectins, which can be utilized by PG. Because the performance of PME is dependent on apoplastic pH, the activity of enzymes was measured in different pH regimes. The response of PME activity to NH_4_^+^ treatment showed a similar trend when using as the substrate pectins with a low (8.9%), medium (30%), or high (90%) degree of methylesterification (Supplementary Figure S1). Therefore, only results for the degree of 30% methylesterification were selected for presentation. The activity of PME in the cell walls of NH_4_^+^-treated plants was decreased by more than 60% at pH 5.0 than in the control, but no differences in enzyme activity were observed at pH 6.8 (**Figure 7A**). At pH 8.5, enzyme activity was below the detection level for all utilized pectins (results not presented). The activity of PG was 3 times lower in NH_4_^+^-treated plants than in the controls (**Figure 7B**).

Moreover, enzymes that are able to cross-link wall polymers were analyzed. The protein level of XTH/XET was unchanged during growth on different N sources (**Figure 7E**). The activity of POX in cell walls was 30% increased in plants treated with NH_4_^+^ than in the controls (**Figure 7C**). PAL is the main enzyme in the pathway for the synthesis of phenolic compounds. The activity of PAL was more than twofold higher in tissues of NH_4_^+^-treated plants than in the controls (**Figure 7D**).

**FIGURE 8:**
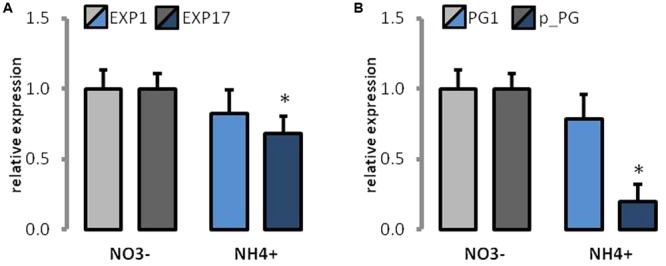
The expression of cell wall modifying proteins in plants grown on NH_4_^+^ and NO_3_^–^ (control). Transcript levels for **(A)** expansions (*EXP1* and *EXP17*), and **(B)** polygalacturonases (*PG1* and *putative PG*). The value for each gene in control plants was set as 1.

The expression of some proteins that are engaged in cell wall loosening was determined. The expression of EXP1 and EXP17 was lower in NH_4_^+^-treated plants (**Figure 8A**). However, only the lowered expression of EXP17 was significant as compared to that of the controls. Further, the expression of both PG was decreased in NH_4_^+^-treated plants (**Figure 8B**), but only the fivefold decrease in the expression of the putative PG protein was significantly altered as compared to that of the control.

### Expression of Cell Wall Dependent Receptor-Like Kinases

**FIGURE 9:**
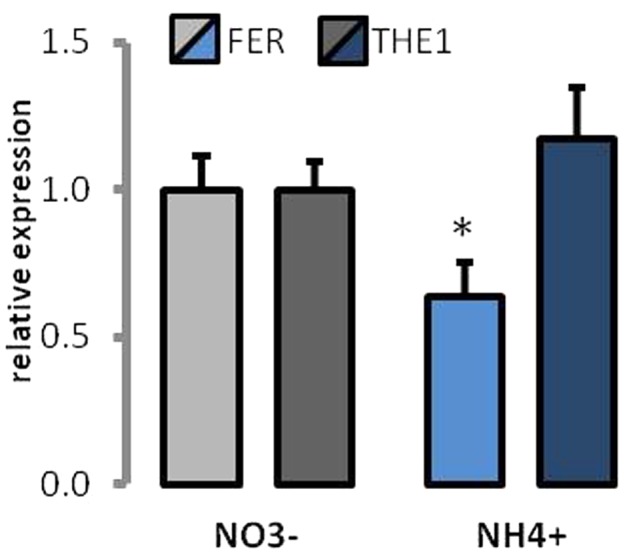
Expression of cell wall receptor-like kinases in plants grown on NH_4_^+^ and NO_3_^–^ (control). Transcript levels for *Thesseus1* and *Feronia*. The value for each gene in control plants was set as 1.

The expression of two of the cell wall sensor receptor-like kinases was determined. *FER* is thought to be associated with regulation of signaling during cell elongation. Approximately 40% lower expression of *FER* was determined in plants growing on NH_4_^+^ as the source of N (**Figure 9**). THE1 is called a cell wall integrity sensor kinase. The expression of *THE1* was unchanged between different N-source treatments (**Figure 9**).

## Discussion

Ammonium restricts expansion and growth of *Arabidopsis* leaves (**Figure 1A**), similar to that in many other plant species (Gerendás et al., 1997; Britto and Kronzucker, 2002). Changes in the size of plant organs may be the result of individual cell growth repression. From our experiments, it was evident that the smaller leaves of NH_4_^+^-treated plants (**Figure 1B**) have smaller cells (**Figure 2B**). The inhibition of cell growth is usually caused by changes in their cell wall properties, which do not allow the cells to expand. These changes include modifications in cell wall composition and/or spatial pattern of the elements, and thus in the mechanical properties of cell wall (Cosgrove, 2005; Benatti et al., 2012). In fact both the higher stiffness and tensile strength of *Arabidopsis* leaf tissues in plants using NH_4_^+^ as the only N source was demonstrated (**Figures 1D,E**). This parameter indicates resistance of the leaf structure to stretching forces that is generally determined by the cell wall properties, turgor and the leaf thickness on the cross-section. The growth rate of elongating cells is often related to the creep capacity, i.e., to the irreversible deformation owing to turgor pressure. Our results indicate that cell walls of NH_4_^+^-treated plants are stiffer, probably due to more intense interlinking of load-bearing wall polymers. Increased mechanical strength of leaf tissues and their cell wall stiffness depend usually on rise in the accumulation of cell wall forming material and/or its components (Keegstra, 2010). The cell walls of NH_4_^+^-treated plants seem thinner (**Figure 2D**). However, when we compared the cell wall thickness to cell size, we observed the opposite effect. The cell wall thickness (**Figure 2D**) in relation to cell size (**Figure 2B**) of NH_4_^+^-treated plants increased as compared to the controls grown on NO_3_^–^. This in turn indicates that the increased mechanical properties of cell walls may be the result of a thicker cell wall layer for each plant cell.

In addition, mechanical properties of cell walls depend on the assembly of cellulose microfibrils, in addition to the cell wall thickness. The microfibrils in the leaves of NH_4_^+^-treated plants showed a more regular aligned pattern than plants grown on NO_3_^–^, where the microfibrils were randomly interwoven (**Figure 3**). Moreover, the microfibrils in the cell walls of NH_4_^+^-treated plants were denser than in those of the NO_3_^–^-treated controls, which might affect their mechanical properties. The orientation and assembly of microfibrils determines the extent to which the cell walls can expand (Dixit and Cyr, 2004; Marga et al., 2005; Oda, 2015). Therefore, the tightly packed cellulose net may affect growth of NH_4_^+^-treated plants. Zhu et al. (2006), when analyzing genes induced by NH_4_^+^ treatment, noticed the induction of cellulose synthase.

The cellulose microfibrils are held together mainly by hemicelluloses, thereby strengthening the cell wall (Scheller and Ulvskov, 2010). The spectral analysis of FTIR suggested that plants grown on NH_4_^+^ as N source may have more xyloglucan in their cell walls (**Figure 5H**), but these results were not statistically relevant. However, immunostaining of xyloglucan, the main polymers of hemicelluloses, in leaves showed an increased incorporation of these polysaccharides in the cell walls of NH_4_^+^-treated plants (**Figures 6B,C**). Similarly, a marked increase in xyloglucan level was observed in the epidermis of the petiole of NH_4_^+^-treated plants (**Figure 6A**). The higher assembly of xyloglucan in cell walls of NH_4_^+^-treated plants could strengthen the structure of the cell wall and withstand tensile stress.

In general, the leaves of NO_3_^–^- and NH_4_^+^-treated plants differed in the spectral features related to polysaccharides. Experimental evidence of alterations in cell wall composition and metabolism in response to NH_4_^+^-treatment are scarce. A lower pectin and hemicellulose content was reported in the root cell walls of two different rice cultivars grown on NH_4_^+^ (Wang et al., 2015). However, it was suggested that these changes in polysaccharide composition under the different N sources tested might be the result of changes in growth medium pH, because buffering the solutions resulted in unchanged pectin and hemicellulose content in these plants. In accordance with the findings mentioned above, our experiments (where the medium pH was 6.5) revealed that the composition of major polysaccharides was unchanged. Even though the content of major polysaccharides in cell wall was the same in the different N treatment regimes, their assembly and localization pattern in cell walls were different.

The cell wall properties depend not only on the amount or pattern of different polysaccharides constructing the cell wall formation but also on their cross-linking, in which cell wall modifying enzymes are engaged. Cessation of cell enlargement involves multiple processes, including tightening of the matrix-cellulose network. The XTH/XETs play a role in dissection and disassembly of hemicelluloses in the cell wall. The activity of XTH/XET cross-links cellulose and hemicelluloses, but in our experiments XTH/XET protein level in cell walls remained unchanged between the N sources (**Figure 7E**). However, the activity of XTH/XET is only significant in growing cells (Fry, 2004). Therefore, during long-term NH_4_^+^ treatment, it may no longer be necessary to strengthen the cell walls, because their construction is already fixed.

Further, cell wall extensibility depends on the action of cell wall loosening proteins. Expansins are responsible for the dissociation of hydrogen bonds between polysaccharides and appear to be the primary wall-loosening proteins (Cho and Cosgrove, 2000; Cosgrove, 2000). We found that the expression of two *Arabidopsis* expansins was decreased during long-term NH_4_^+^ growth (**Figure 8A**). Although their activity remains to be demonstrated, the lower expression of some EXP in plant growing on NH_4_^+^ as the only N source may be one of the major factors limiting cell growth. The similar occurrence of a cell wall-associated response was found in a microarray-based study by Patterson et al. (2010), where the cell-wall loosening EXPs and enzymes, such as PGs, endotransglucosylases, and pectate lyases, were down-regulated in response to the short-term NH_4_^+^ treatment of *Arabidopsis* plants.

In addition to the cellulose-hemicellulose network, the pectin matrix plays a critical role in determining the elasticity of the primary cell wall (Peaucelle et al., 2012). Although we did not observe marked changes in total pectin content during the NH_4_^+^ treatment (**Figure 4B**), and FTIR analysis did not reveal any differences in their degree of methylesterification (**Figure 5G**), we found differences in spatial arrangement of methylated pectins (**Figure 6**), which accumulated in the cell walls of epidermal and mesophyll leaf cells. Moreover, for pectin gel formation, the activity of PMEs can be of importance. When PMEs act randomly on pectic polymer, this de-methylesterification promotes the action of PG, leading to pectin hydrolysis. The over-expression of PME inhibitors leads to stiffening of cell walls and the inhibition of organ formation, whereas the over-expression of a PME isoform leads to cell wall softening (Peaucelle et al., 2011). In our experiments, the activity of acidic PME, which is responsible for the degradation of pectins via PG activity (Micheli, 2001), was mostly decreased (**Figure 7A**). Further, the reduced activity and expression of PG (**Figures 7B, 8B**) in plants treated with NH_4_^+^ might lead to rigidification of the pectin matrix. The low PME activity was in accordance with an accumulation of methylated pectins in leaf blades as observed by immunostaining with LM20 antibody (**Figures 6B,C**). Meanwhile, no differences in the distribution of de-methylated pectins, observed by immunostaining with LM19 antibody were detected (**Figures 6B,C**). A low level of pectin methyl-esterification is often associated with reduced cell wall extensibility and reduction in growth (Derbyshire et al., 2007; Pelletier et al., 2010). Another possibility is that when neutral and basic PMEs act linearly to produce free carboxyl groups cooperative calcium binding of contiguous carboxyl groups on two adjacent pectin chains can lead to cell wall stiffening. However, the content of Ca^2+^ ions in tissues of plants grown on NH_4_^+^ was decreased (**Figure 4F**). The lower activity of neutral PME might release lesser number of binding sites containing negatively charged galacturonic acid for Ca^2+^ binding. It should be noted that the content of Ca^2+^ was measured in whole leaf tissues, and therefore does not exactly correspond to cell wall levels. Moreover, it was proposed that the major factor determining Ca^2+^ distribution in plant tissues is the expression of the calcium exchangers (CAXs) (Hirschi, 1999; Conn et al., 2011). The dysfunction of the transporters CAX1 and CAX3 in double mutants resulted in disturbed import of Ca^2+^ into the vacuole, which resulted in Ca^2+^ accumulation in the apoplast (Conn et al., 2011). In a microarray-based study, it was shown that NH_4_^+^ supply specifically triggers down-regulation of the expression of *CAX1* and *CAX3* genes (Patterson et al., 2016). This suggests that the NH_4_^+^ mediated changes in Ca^2+^ distribution could be related to lower activities of CAX transporters and lead to Ca^2+^ accumulation in the apoplast, even though tissue Ca^2+^ levels was decreased (**Figure 4F**). An increased content of Ca^2+^ in the apoplast during NH_4_^+^ nutrition could possibly induce the cross-linking of pectins by Ca^2+^ and lead to stiffening of the cell wall. To clarify this apparent contradiction in ion location single cell analysis is necessary. Further, pectins can be cross-linked through borate-diol esters, which can be formed between the apiofuranosyl residues of the side chains of rhamnogalacturonan-II (RG-II). In our studies on ion spectrum, the content of boron was increased (**Figure 4G**). The slightly higher portion of neutral sugars in the pectin-enriched fraction may suggest that plants have more rhamnogalacturonans in cell walls during the NH_4_^+^ treatment. It would be interesting to analyze the monosaccharide content in cell walls in order to determine if a higher RG-II level can be responsible to create more borate diester bonds in cell walls of NH_4_^+^ grown plants. This could compensate for the low binding of Ca^2+^ to pectins. Furthermore, it was proposed that cell wall thickness depends on the availability of boron ions. Boron-deficient plants had swollen cell walls, whereas boron-treated plants resulted in thin cell walls (Ishii et al., 2001). O’Neill et al. (2001) showed that the reduced stem growth and tensile strength of the *mur1* mutant is reversed to near wild type levels by spraying plants with excess boron.

The activity of POX may lead to decreased cell wall extensibility by the formation of diphenyl bridges among cell wall polymers, such as hydroxyproline-rich glycoprotein, pectins, or hemicelluloses. POXs were found to participate in the cross-linking of polymers by the oxidation of a wide variety of small phenolic compounds (Czaninski et al., 1993; Marjamaa et al., 2009; Ros Barceló and Gómez Ros, 2009). Similarly, the content of cell wall phenolics (the substrates for POX) were elevated during growth of plants using NH_4_^+^ as the only N source (**Figure 4E**), and the main enzyme responsible for phenolics production (PAL) showed an increased activity (**Figure 7D**). As a co-substrate, POX utilizes H_2_O_2_. An increased H_2_O_2_ level was observed in the cell walls under long-term NH_4_^+^ treatment (Podgórska and Szal, 2015; Podgórska et al., 2015), which might stimulate the activity of POX. Moreover, elevated cell wall POX activities and H_2_O_2_ production were observed in the roots during NH_4_Cl treatments of rice seedlings, which were presumed to regulate growth (Lin and Kao, 2001). Hence, an increased apoplastic POX activity (**Figure 7C**) may strengthen the cell walls and promote inhibition of cell expansion when grown on NH_4_^+^ as the only N source. Notably, POX activity might be involved in cell wall stiffening, in addition to the antagonistic processes, such as wall loosening. In the hydroxylic cycle, POX can produce HO^.^ from the superoxide anion and hydrogen peroxide (Liszkay et al., 2003; Passardi et al., 2004). The HO^.^ radical may cause non-enzymatic cell wall loosening, which enables growth (Cosgrove, 2005; Ros Barceló and Gómez Ros, 2009). Because the amount of H_2_O_2_ deposition in cell walls of NH_4_^+^ grown plants is increased (Podgórska and Szal, 2015), the accumulation of HO^.^ may be enhanced.

In some tissues, plants can build a secondary cell wall, which is a thicker additional layer of cellulose and may contain lignin. Lignification is a temporal process in which phenolic polymers are deposited in the cell walls, which may increase wall rigidity (Barros et al., 2015). The major enzymes responsible for this process are POX and laccases. As reported above, the higher POX activity (**Figure 7C**) together with elevated cell wall phenolic content (**Figure 4E**) when grown on NH_4_^+^ may indicate the occurrence of cell wall lignification. Even though total lignin quantification in *Arabidopsis* leaves did not show any significant differences (**Figure 4D**), the analysis of the FTIR spectrum indicated an elevated phenolic content and contribution of lignifications in cell walls of NH_4_^+^-nourished plants. These results point to the initiation of the lignifications process in *Arabidopsis* leaves, even though in our experiments, relatively young leaves were analyzed, where lignifications is usually not a major process. Relatively earlier cessation of cell expansion in the NH_4_^+^-treated plants than in the NO_3_^–^-treated plants may partially explain the observed phenotypic dissimilarities.

Another possibility how cell wall remodeling can be accomplished is through altered endocytosis. The process of endocytosis involves mainly the coordinated transport of plasmalemma proteins from one area of the membrane to another (or degradation in the vacuole) (Russinova et al., 2004; Dhonukshe et al., 2006), but in these endocytic vesicles also cell wall polysaccharides can be transported, including boron-cross linked RG-II, pectins and xyloglucan (Baluška et al., 2002, 2005). It was shown that endocytic vesicles carry the PIN2 auxin efflux carrier proteins, which are responsible for polar auxin transport and consequently adequate gravitropism. However, in NH_4_^+^ grown plants the process of gravitropism was impaired, which was related to the lack of PIN2 (Zou et al., 2013). Therefore, it can be expected that endocytosis may also be impaired during NH_4_^+^ nutrition and thereby may also affect the assembly of cell walls, which needs further research though. Moreover, changes in the availability of cell wall binding ions can affect endocytosis, boron deprivation was shown to inhibit internalization of pectins (Yu et al., 2002).

The plant cell wall actively participates in receiving signals from the environment and coordination of plant growth (Wolf et al., 2012; Tenhaken, 2014; Le Gall et al., 2015). In this signaling process, the *Catharanthus roseus* receptor-like kinases (CrRLKs) are involved, among which the most prominent members are *Theseus1* (*THE1*), *Feronia* (*FER*), and *Hercules* (*HER1 and HER2)*. CrRLKs are plasmalemma-bound proteins that were proposed to have potential extracellular motifs, which can interact with cell wall components (Cheung and Wu, 2011; Wolf and Höfte, 2014; Li et al., 2016). The expression of all these CrRLK is high in elongating tissues and their function was proposed to be related to the regulation of expansion growth (Guo et al., 2009; Lindner et al., 2012; Nissen et al., 2016). The activation of THE1 signal transduction in a *prc1* mutant with a deficiency of cellulose synthesis is responsible for the inhibition of cell elongation (Hématy et al., 2007). Therefore, the role of THE1 in cell-wall-integrity signaling pathway and growth regulation was proposed. The unchanged expression of THE1 in the NH_4_^+^-treated plants (**Figure 9**) may imply that cell wall deposition in these plants is not affected, which correlates with unchanged levels of major polysaccharides (**Figures 4A,B**). However, FER participates in many physiological processes involved in plant growth and reproduction (Guo et al., 2009). The first FER discovered was found to trigger the cessation of pollen tube growth (Huck et al., 2003; Escobar-Restrepo et al., 2007). In addition, FER may function as a receptor for polar growth (Kanaoka and Torii, 2010), and the expression of FER can promote reactive oxygen species-mediated root hair development (Duan et al., 2010). In general, FER is essential for expansion and growth, and the defection of this CrRLK in mutant plants triggers a growth-inhibited phenotype. Therefore, we expect that the lower expression of FER in response to NH_4_^+^ treatment (**Figure 9**) may be connected with disturbed cell growth.

## Conclusion

The present work provides the first direct evidence that the use of NH_4_^+^ as the only N source leads to cessation of cell enlargement that corresponds with cell wall rigidity. Adaptive plasticity of the cell wall during NH_4_^+^ nutrition is manifested by tightening of the matrix-cellulose network that provide elevated mechanical strength. The main factors in the process of cell wall stiffening may involve the decreased expression and/or activity of expansins and cell wall hydrolyzing enzymes (PME, PG) or increased activity of cell wall cross-linking enzymes, such as POX. These NH_4_^+^-mediated changes in cell wall assembly and metabolism may retard growth of plants.

## Author Contributions

AP designed the experiments, measured the content of different cell wall components, performed TEM and CLSM microscopy. AP and MB measured enzyme activities and carried out RT-PCR analysis. AP and JZ measured leaf tensile stiffness. JZ carried out FTIR analysis. KG performed AFM measurements. MO-B prepared samples for ion determination. AP wrote the manuscript. BS and DS revised the manuscript.

## Conflict of Interest Statement

The authors declare that the research was conducted in the absence of any commercial or financial relationships that could be construed as a potential conflict of interest.
