# Supplementary figures and images for: Altered Cell Wall Plasticity Can Restrict Plant Growth under Ammonium Nutrition
Source: Front Plant Sci. 2017 Aug 10;8:1344. doi: 10.3389/fpls.2017.01344 (PMC5554365; doi:10.3389/fpls.2017.01344)

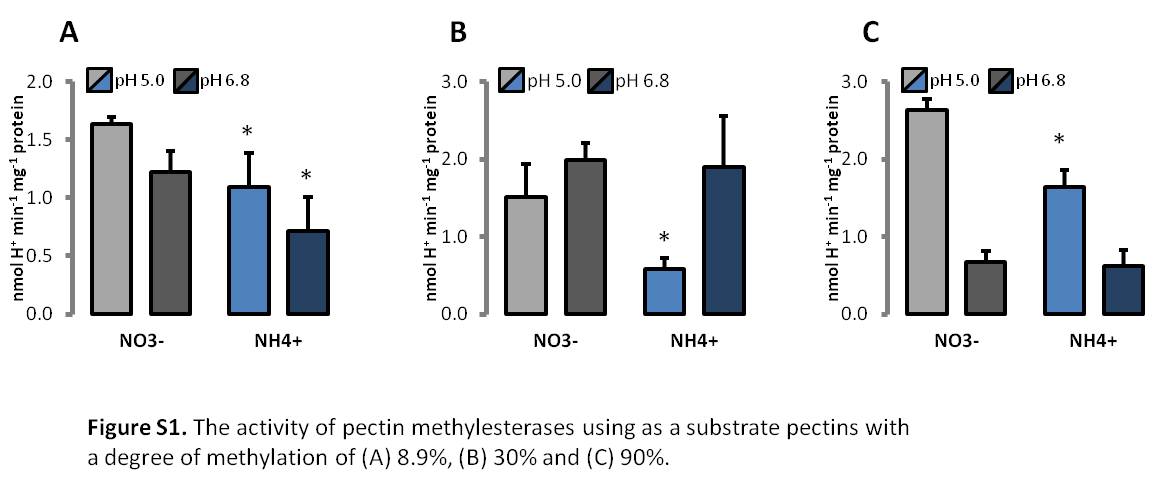

Supplement: Supplementary file 1 [file Image_1.JPEG]
